# Men Who Have Sex with Men (MSM) and Factors Associated with Not Using a Condom at Last Sexual Intercourse with a Man and with a Woman in Senegal

**DOI:** 10.1371/journal.pone.0013189

**Published:** 2010-10-05

**Authors:** Joseph Larmarange, Abdoulaye S. Wade, Abdou K. Diop, Oulimata Diop, Khady Gueye, Adama Marra, Annabel Desgrées du Loû, for ELIHoS Group

**Affiliations:** 1 CEPED (Centre Population & Développement - UMR 196 - Paris Descartes/INED/IRD), IRD (Institut de Rechercher pour le Développement), Paris, France; 2 Division SIDA/IST, Institut d'Hygiène Sociale, Dakar, Sénégal; 3 US009, IRD (Institut de Recherche pour le Développement), Dakar, Sénégal; 4 Laboratoire de Virologie, Hôpital Le Dantec, Dakar, Sénégal; 5 Université Cheikh Anta Diop, Dakar, Sénégal; Stanford University, United States of America

## Abstract

**Background:**

Men who have sex with other men (MSM) are a vulnerable population in Africa that has been insufficiently explored. Given the high rate of bisexuality among MSM (73% in the past year), it is important to understand their risk-taking behaviors regarding both men and women.

**Methodology/Principal Findings:**

A socio-behavioral survey was carried out in 2007 among 501 MSM recruited using the snowball sampling method. We explore in this article why a condom was not used during last sexual intercourse with a man and with a woman, taking into account the respondent's characteristics, type of relationship and the context of the sexual act. In the survey, 489 men reported that they had had sexual intercourse at least once with another man during the previous year, and 358 with a man and with a woman. The main risk factors for not using a condom at last sexual intercourse with another man were having sex in a public place (aOR = 6.26 [95%CI: 2.71–14.46]), non-participation in an MSM prevention program (aOR = 3.47 [95%CI: 2.12–5.69]), a 19 years old or younger partner (aOR = 2.6 [95%CI: 1.23–4.53]), being 24 years or younger (aOR = 2.07 [95%CI: 1.20–3.58]) or being 35 years or over (aOR = 3.08 [95%CI:1.11–8.53]) and being unemployed (aOR = 0.36 [95%CI: 0.10–1.25]). The last sexual intercourse with the respondent's wife was hardly ever protected (2%). With women, the other factors were a 15 years or younger partner (aOR = 6.45 [95%CI: 2.56–16.28]), being educated (primary: aOR = 0.45 [95%CI: 0.21–0.95], secondary or higher: aOR = 0.26 [95%CI: 0.11–0.62]), being a student (aOR = 2.20 [95%CI: 1.07–4.54]) or unemployed (aOR = 3.72 [95%CI: 1.31–10.61]) and having participated in a MSM prevention program (aOR = 0.57 [95%CI: 0.34–0.93]).

**Conclusion:**

Having participated in a prevention program specifically targeting MSM constitutes a major prevention factor. However, these programs targeting MSM must address their heterosexual practices and the specific risks involved.

## Introduction

In Sub-Saharan Africa, HIV transmission was described from the start as an essentially heterosexual problem, and to a lesser extent, as a perinatal problem [1]. The question of homosexual HIV transmission was ignored for many years. Although the existence of homosexual practices on the African continent has long been reported [2], the first epidemiological study to measure HIV prevalence among men having sex with men (MSM) was only conducted in 2004 in Senegal [3]. Since then, several studies carried out in various African countries have confirmed that HIV prevalence among MSM is between 2 to 20 times higher than among the general population [4]–[6].

As within other continents, African MSM are particularly vulnerable to HIV, especially since in most African countries homosexuality is highly stigmatized; it may constitute a crime and penal sanctions can even entail the death penality [7], [8]. Men who have sex with other men very often conceal their homosexuality from their families and friends, and avoid seeking health care through fear of rejection [9]–[12]. HIV prevention programs are insufficiently taking his specific group into consideration: only 27 countries out of 86 (31%) included MSM in their national HIV surveillance report in 2007 [13].

In Senegal, the study conducted in 2004 showed HIV prevalence among MSM to stand at 21.5%, i.e. 30 times higher than among the general population [3]. Thereafter, the health authorities in Senegal, together with certain NGOs, set up programs specifically targeted towards men having sex with other men: a specific program for STI and HIV management, involving health professionals trained to give support and care to this stigmatized population; an awareness campaign about sexual risks within MSM circles; and an appeal to all public figures to urgently address the specific risks incurred by MSM. An important political argument justifying the implementation of these programs, in a society where homosexuality is condemned, is the protection of the population as a whole: if the epidemic within the homosexual population is not addressed, the efforts in Senegal to control AIDS could be jeopardized, since the epidemic can spread to the entire population through the heterosexual practices of these men.

Yet, little research in Senegal has studied in detail the heterosexual practices of MSM, even though this aspect has been mentioned in the literature. N. Teunis [11] reveals that certain MSM are married or have “girlfriends”. R. Sappe also mentions this bisexual behavior, but reduces it to a heterosexual relationship providing a “social cover” [14]. An ethnographic survey conducted in 2000–2001 in Dakar estimated that 88% of the 250 MSM respondents declared having had vaginal intercourse with a woman at least once during their lifetime [9]. The epidemiological study conducted in 2004 confirmed this high proportion of bisexual practices: 94% of the MSM surveyed had already had sexual intercourse with a woman during their lifetime, and 74% during the previous twelve months [3].

If prevention programs are to be more effective, it is critical to understand the factors behind MSM risky behavior, not only with their male partners, but also with their female partners. We present here an analysis of factors for not using a condom at last sexual intercourse with a man and with a woman, using data from a new epidemiological and behavioral survey carried out in 2007 among men having sex with men in Senegal (the ELIHoS survey). Individual characteristics, characteristics of the relationship and context of the sexual act were included in the analysis.

## Methods

The ELIHoS survey (Evaluation of campaigns targeting male homosexuals in Senegal – ANRS 12139) was conducted in 2007. The main objectives of this survey were to measure HIV and STI (sexually transmitted infection) prevalence, and sexual behaviors of MSM, in order to observe trends since 2004 (previous survey), and thus evaluate the effects within this population of prevention campaigns currently underway. This survey was initiated by the AIDS-STI division of the Institut d'Hygiène Sociale in Dakar, with the full agreement of the Senegalese Ministry of Health.

The recruitment method used was the “snowball sampling” technique. MSM peer leaders were in charge of recruiting participants in three places (Dakar, the capital city; Mbour/Thiès, two towns near the tourist coast; Saint-Louis, fairly large town in northern Senegal). Recruitment was carried out in various types of place (bars and nightclubs where MSM meet up, MSM associations, brothels frequented mostly by MSM, certain beaches) and by word of mouth in various social settings. No financial incentive was offered to respondents other than reimbursement of transport costs (10 000 F CFA). On the other hand, MSM peer leaders were remunerated for their participation in the research.

Recruitment criteria were age (18 years or over) and history of sexual intercourse with other men. The survey was presented as a survey into the specific health needs of MSM (including HIV and STI). The survey was conducted in the health units set up in 2003 dedicated to the medical care of MSM, and where respondents knew that they would be made welcome and where total confidentiality was ensured. The men who agreed to participate in the survey went along to these health units where they were interviewed by a doctor and a social worker who explained survey objectives and procedure. After having signed a consent form, they answered a socio-behavioral questionnaire administered by the social worker or by the doctor. Thereafter the doctor carried out a clinical examination and took samples of blood and urine for biological analysis. If an STI was found, a syndromic treatment was prescribed. The respondent was asked to return two weeks later to pick up the laboratory test results. When he came back, any infection that had been detected was treated. All the treatments were delivered free of charge. If the respondent tested HIV-positive, he was informed by the social worker who had been trained to give this information. He was then taken in hand by the HIV management program where a full biological analysis was carried out in order to decide whether or not he would receive antiretroviral treatment (treatment is free of charge in Senegal). The entire survey guaranteed that respondents would remain strictly anonymous. Questionnaires, consent forms and clinical and biological data were matched using numbered stickers (the numbers were allotted to respondents so that they could collect their laboratory results). The investigators were trained to fully apply this rule of confidentiality.

The epidemiological results of this survey have been published in another paper [15]. We analyze here the factors related to unprotected penetration during last sexual intercourse with a man and during last sexual intercourse with a woman, in the last year before the survey. Only men having had at least one intercourse with a man during the last year before the survey have been considered for the analysis of the last sexual intercourse with a man. Among them, only men having had at least one intercourse with a woman during the last year before the survey have been considered for the analysis of the last sexual intercourse with a woman.

Bivariate associations were determined using Wald test for bivariate logistic regression. Three groups of variables were included in the analysis: respondent characteristics at the time of the survey (survey site, age group, education level, occupation, awareness of HIV, sex of sexual partners during the last year before the survey, sex of regular partners at the time of the survey); type of relationship with the partner of last sexual intercourse (partner type, loving relationship, length of relationship, partner's age, age difference with the partner); and variables regarding the context of last sexual intercourse (where it took place, awareness of HIV status of partner, payment for this sexual act).

The ‘occupation’ variable distinguishes students and apprentices from individuals receiving salary. For the latter, the common professions among Senegalese MSM were grouped together. These were hairdressers, beauticians, dressmakers, tailors, weavers, artists, photographers, models, bar tenders, waiters and people working in tourism.

Three variables allow the levels of information, awareness of and participation in an HIV prevention program to be assessed: knowledge about HIV counseling and testing centre, participation in at least one prevention program specifically targeting MSM, and membership of an MSM association. Since these variables overlapped, we created a global awareness indicator to account for the following: people unaware of HIV counseling and testing centre location; those aware of an HIV counseling and testing centre location but never having participated in a prevention campaign; those aware of an HIV counseling and testing centre location and having participated in a prevention campaign but not members of an association; and those meeting all three requirements.

In order to avoid chance selection, a first multiple logistic regression model with all factors with p<0.20 in the bivariate analysis was calculated in order to merge modalities of the same variable with similar odds ratio. Then, new models using merged variables were calculated through a step-by-step backwards elimination procedure. Finally, the model with the lowest AIC (Akaike's Information Criterion) was retained [16], [17].

Questionnaires were entered using Microsoft Access, and statistical analysis was carried out using SPSS 16.0 (SPSS Inc; Chicago, Illinois).

### Ethics Statement

The national ethics committee of Senegal gave its ethical approval to the study, which was subject to an ethical audit by the Agence Nationale de Recherche sur le SIDA.

## Results

The socio-behavioral questionnaire was administered to 501 men (306 men in Dakar, 100 in Mbour/Thiès and 95 in Saint-Louis). The sample was young (80% were under thirty) and fairly well-educated (47% had reached secondary school level or higher education). Ninety per cent lived with their family, and 41% declared themselves to be members of an MSM association.

Six men (1.2%) declared that they had had no sexual intercourse over the last twelve months, and six others had only had female partners during this period. These twelve men were excluded from the analysis. Three hundred and fifty-height respondents (73%) out of the 489 remaining men had had both male and female partners over the past year. As for last sexual intercourse with a man, anal sex was nearly universal (see Table 1): only two men had not practiced anal sex and only oral-penile sex without use of a condom. Oral-penile sex was almost always unprotected, and neither was oral-anal sex which remained uncommon (5% of last sexual intercourse). Regarding last sexual intercourse with a woman, three men declared no vaginal penetration: one declared oral-penile sex, two others declared anal penetration. This latter practice remained rare with women (1%). As with men, oral-penile sex with women was unprotected.

**Table 1:** Sexual practices at last sexual intercourse.

Practices at last sexual intercourse		frequency		unprotected	
		%	n	%	n
With a man					
	Anal sex (insertive or receptive)	99.6	487	23.2	113
	- insertive anal sex	50.9	249	24.9	62
	- receptive anal sex	52.6	257	21.4	55
	Oral-penile sex	24.9	122	90.2	110
	Oral-anal sex	4.9	24	83.3	20
	Total		489		
With a woman					
	Vaginal sex	99.2	355	37.5	133
	Anal sex	1.1	4	75.0	3
	Oral-penile sex	7.0	25	100.0	25
	Total		358		

Sum of columns exceeds 100% (several answers possible).

Regarding last sexual intercourse with a man (Tables 2, 3 and 4), there was a strong association between lack of condom use and the place of the sexual intercourse: 65% of sexual acts that took place in a public place or outside (park, beach, cinema, bar or nightclub toilet) were unprotected, compared with between 16–21% in other places. When controlling for other factors, the odds ratio for not using a condom was about six times higher when the intercourse took place outside compared to within the respondent's home (aOR = 6.26 [95%CI: 2.71–14.46). Respondents are too few (n = 34) to assess if other factors come into play when sexual intercourse takes place outside.

**Table 2:** Unprotected anal or vaginal sex at last sexual intercourse with a man and at last sexual intercourse with a woman (bivariate analysis).

Last sexual intercourse		with an man^a^			with an woman^b^		
		%	n/N	p-value^c^	%	n/N	p-value^c^
**Respondent characteristics**							
Site				0.070			0.606
	Dakar	23.6	71/301		35.8	77/215	
	Saint-Louis	29.7	27/91		39.0	30/77	
	Mbour/Thiès	15.5	15/97		42.4	28/66	
Age group				0.001			0.212
	18–19 years	36.5	35/96		38.9	28/72	
	20–24 years	24.3	46/189		36.4	47/129	
	25–29 years	13.5	15/111		34.6	27/78	
	30–34 years	12.3	8/65		33.3	18/54	
	35 years or over	32.1	9/28		60.0	15/25	
Education				0.219			0.002
	None	29.0	20/69		62.0	31/50	
	Primary	18.7	36/193		33.1	45/136	
	Secondary	25.9	50/193		35.6	53/149	
	Higher	20.6	7/34		26.1	6/23	
Occupation				0.018			0.170
	None	37.0	10/27		55.0	11/20	
	Student/Apprentice	29.4	37/126		37.8	34/90	
	Frequent profession among MSM	16.7	18/108		32.4	23/71	
	Other profession	21.1	48/228		37.9	67/177	
Global awareness indicator				0.000			0.004
	Don't know a place to be tested for HIV	40.8	31/76		53.3	32/60	
	Know a place to be tested for HIV (a)	35.7	40/140		43.8	46/105	
	(a) + has participated in an MSM program (b)	15.1	14/93		28.8	19/66	
	(a) + (b) + is member of an MSM organization	10.0	27/114		29.9	38/127	
Sexual partners last year				0.367			na
	Men exclusively	26.0	34/131		na	na	
	Men and women	22.1	79/358		37.7	135/358	
Regular partners at the survey				0.608			0.844
	None	23.5	20/85		40.0	20/50	
	Women	29.4	15/51		41.2	21/51	
	Men	24.0	35/146		40.0	20/50	
	Men and women	20.8	43/207		35.7	74/207	

Continue in Table 3.

**Table 3:** Table 2 continued.

Last sexual intercourse		with an man^a^			with an woman^b^					
		%	n/N	p-value^c^	%	n/N		p-value^c^		
**Relationship characteristics**										
Type of partner				0.686				0.000		
	Regular partner	22.5	64/285		30.1	56/186				
	Occasional partner	24.0	49/204		36.6	53/145				
	Spouse	na	na		96.3	26/27				
Loving relationship with this partner				0.455				0.412		
	No	24.7	55/223		35.9	47/131				
	Yes	21.8	58/266		38.8	88/227				
Duration of the relationship				0.670				0.250		
	First time with this partner	22.1	31/140		42.1	48/114				
	Less than 3 months	18.6	13/70		23.5	8/34				
	3 to 12 months	23.0	23/100		35.0	28/80				
	One year or more	25.7	46/179		39.2	51/130				
Age of the partner				0.001				0.001		
	15 years or under	57.1	4/7		77.4	24/31				
	16–19 years	40.0	20/50		35.7	55/154				
	20–24 years	26.8	40/149		33.7	32/95				
	25–29 years	16.4	23/140		31.8	14/44				
	30 years or over	18.3	26/142		29.4	10/34				
Age difference with this partner				0.551				0.003		
	Partner younger by 10 years or more	20.0	4/20		58.6	34/58				
	Partner younger by 2 to 9 years	20.0	16/80		37.7	69/183				
	Same age (± one year)	27.6	37/134		29.1	23/79				
	Partner older by 2 to 9 years	23.4	46/197		24.1	7/29				
	Partner older by 10 years or more	17.5	10/57		22.2	2/9				
**Context of sexual act**										
Location of sexual act				0.000				0.670		
	Respondent's home	20.1	20/149		36.3	85/234				
	Partner's home	21.2	56/264		43.9	25/57				
	Public place/Outside	64.5	20/31		50.0	3/6				
	Hotel/A friend's home	15.6	7/45		36.1	22/61				
Partner's HIV status known				0.014				0.296		
	No	24.7	110/445		37.3	131/351				
	Yes	6.8	3/44		57.1	4/7				
Payment for this sexual act				0.077				0.268		
	No	26.5	78/294		39.5	115/291				
	Yes, money given by respondent	21.9	7/32		27.8	15/54				
	Yes, money received by respondent	17.2	28/163		38.5	5/13				
**All**		23.1	113/489		37.7	135/358				

a Base: 489 MSM who had had at least one male sexual partner over the past year.

b Base: 358 MSM who had had both male and female sexual partners over the past year.

c Wald test for bivariate logistic regression of the overall variable (null hypothesis: all odds ratio of modalities are equal to 1).

na: not applicable.

**Table 4:** Factors associated with unprotected anal or vaginal sex at last sexual intercourse with a man and at last sexual intercourse with a woman (logistic regression).

		aOR	95% CI	p-value (Wald test)
**Last sexual intercourse with a man** a				
Location of sexual act:	public place/outside	6.26	2.71–14.46	0.000
Has participated in a MSM prevention program		0.28	0.18–0.46	0.000
Partner is 19 years or younger		2.36	1.23–4.53	0.010
Age of the respondent	18–24 years	2.07	1.20–3.58	0.009
	25–34 years	1		—
	35 years or over	3.08	1.11–8.53	0.030
Is unemployed		2.81	1.10–7.19	0.031
Partner's HIV status known		0.36	0.10–1.25	0.107
**Last sexual intercourse with a woman** b				
Partner is the spouse		68.75	8.76–539.62	0.000
Partner is 15 years or younger		6.45	2.56–16.28	0.000
Education of the respondent	None	1		—
	Primary	0.45	0.21–0.95	0.037
	Secondary or Higher	0.26	0.11–0.62	0.002
Occupation of the respondent	Has a job	1		—
	Student/Apprentice	2.20	1.07–4.54	0.032
	Unemployed	3.72	1.31–10.61	0.014
Has participated in a MSM prevention program		0.57	0.34–0.93	0.024

a Base: 489 MSM who had had at least one male sexual partner over the past year.

b Base: 358 MSM who had had both male and female sexual partners over the past year.

aOR: adjusted Odds Ratio. 95% CI: 95% Confidence Interval.

The second factor strongly related to not using a condom was the level of information and awareness concerning HIV: proportion of unprotected sex at last sexual intercourse was 41% among individuals with no knowledge of an HIV counseling and testing centre, compared to 10% among MSM association members having participated in a prevention campaign (p<0.001). In particular, having participated at least once in an HIV counseling and testing program specifically targeting MSM was associated with a lesser likelihood of unprotected sex (aOR = 0.28 [95%CI: 2.71–14.46]).

Probability for not using a condom was significantly (p<0.05) higher when the male partner was young (19 years or under), among the younger age group (18–24 years) and among the older age group (35 years and over), and also among unemployed individuals.

Finally, although not significant (p = 0.107), it seemed to be half as high when the serological status of the partner was known.

Concerning last sexual intercourse with a woman, sexual acts with the respondent's wife were hardly ever protected (26 out of 27 times). Secondly, lack of condom use was almost six times higher (aOR = 6.45 [95%CI: 2.56–16.28]) when the female partner was very young (15 years or younger).

Unprotected sex with a woman was more commonly declared by students and apprentices (aOR = 2.20 [95%CI: 1.07–4.54]) and unemployed people (aOR = 3.72 [95%CI: 1.31–10.61]). On the contrary, probability for using a condom was higher among educated people (aOR = 0.45 [95%CI:0.21–0.95] for primary level, aOR = 0.26 [95%CI:0.11–0.62] for secondary or higher level) and individuals having followed a prevention program specifically targeting MSM (aOR = 0.57 [95%CI:0.34–0.93]).

## Discussion

The sample used in the ELIHoS survey, as for all samples obtained using the snowball method, incurs a selection bias, since the more “accessible” individuals were more likely to respond to the survey. Thus, for example, only 20% of the surveyed men were 30 years old or older, whereas 51% declared that their sexual partners were 30 years old or older. This is however one of the few recruitment methods possible for use in a survey of a highly stigmatized population and for which no survey base exists. Due to the difficulties inherent to carrying out a survey within this population, our study is the first in Africa to supply information about MSM risk-taking behavior in intercourse with both men and women.

Analysis of last sexual intercourse allows a specific risky behavior to be examined along with the different reasons for this behavior: respondent characteristics along with those of his male or female partner, but also information about their relationship and the location of the sexual acts. Our results show the importance of taking into account the characteristics of the partner and the context of the sexual acts: the main factors for not using a condom with a male partner were the location of sexual intercourse, having not participated in a MSM program and the age of the male partner. With a female partner, the main factors were the type of relationship (spouse or not) and the age of the female partner. It has been long shown that practicing safer sex depends on the cultural context and the relationship involved, and that individual characteristics are not sufficient in explaining a safer approach to sex. Practices can differ depending on the type of relationship with the male or female partner [18], [19], the immediate context of sexual intercourse [20] or the power relations between partners [18], [21].

Contrary to expectations, condom use did not vary, between partnerships with men or women, according to type of relationship (excluding wives): regular or occasional partner, loving relationship or not, established or recent relationship. Also, condom use does not depend on being exclusively homosexual (or not) at the time of the survey, nor by the sex of regular partners. In another article focusing on the various homo-bisexual situations in this survey, we observed that differences regarding risk-taking behaviors, exposure to HIV, and HIV prevalence, were not due to different approaches to condom use connected with type of homo-bisexuality, but were due to frequency of sexual intercourse, type of sexual practices (insertive and/or receptive), and individual involvement in different sexual networks and partnerships, as well as their emotional nature [22].

Condom use by bisexual MSM with women was similar to that observed in the general population of Senegal. According to the DHS report (Demographic and Health Survey) carried out in 2005 among the general population in Senegal [23], 4.0% of men aged between 15 and 49 used a condom during last sexual intercourse with wife or live-in female partner, and 61.9% during last high-risk sexual intercourse (neither wife nor live-in female partner). By comparison, in the ELIHoS survey, the proportion of respondents reported using a condom at last sexual intercourse with wife was 3.7%, and 67.1% at last sexual intercourse with other female partner. Our results were also similar to the DHS results as regards the connection between condom use and education level: in the DHS study, condom use stands at 45% for uneducated men and at 74% for men having reached secondary or higher education levels.

Our results show that not using a condom in heterosexual relationships was higher when the female partner was very young (15 years or younger). According to the anthropological interviews conducted for the ELIHoS project [24], these very young women were mostly considered to be potential future wives. They appeared to be more submissive and less emancipated from a sexual point of view. Thus, men who found themselves obliged to marry due to social pressure, but planned to have a loving relationship with a man at the same time, were liable to choose this type of inexperienced woman and impose their life style. Several studies have shown that women are more vulnerable when they are the dominated party within the couple [25], [21].

Since few years, several papers about quantitative surveys among MSM in sub-Saharan Africa have been published [3], [6], [15], [26]–[32]. All show that bisexual practices are very common among MSM. If most of them provide indicators on unprotected anal intercourse with men [3], [6], [15], [27]–[30], only a survey in South Africa [30] and the two Senegalese surveys [3], [15] provide indicators on unprotected practices of MSM with women. Five articles have analyzed factors associated with HIV positive status [3], [6], [26], [30], [32], but only two have explored factors associated with unprotected anal intercourses [28], [29].

In Cameroon in 2008 [28], significant factors positively associated with having had at least one unprotected anal intercourse in the last six months were: having had at least one stable male partner during lifetime and frequency of sexual intercourses. Although not significant, having not been exposed to HIV prevention interventions was associated with an adjusted odds ratio of 2.04 (95% CI: 0.95–4.35, p = 0.066). In a survey conducted in 2004 and 2005 in a South African Township [29], reporting unprotected anal intercourse with male partners in prior 6 months was significantly and positively associated with regular alcohol use in last month, not using latex-compatible lubrication and rectal trauma.

To our knowledge, no other study on MSM in sub-Saharan Africa has discussed factors associated with not using a condom with women, nor has taken into account the context of the sexual act or characteristics of partner.

In order to guarantee confidentiality during the survey, the socio-behavioral questionnaire did not require the respondent to give information about his supposed HIV serological status. This implies that we couldn't analyze if men knowing their HIV status differed from the others. Several studies have shown that sexual prevention is different between HIV-infected people and people who are seronegative or have an unknown serostatus [33]. In our studies, the proportion of men knowing their status is probably higher among MSM having participated in HIV counseling and testing program specifically targeting MSM and among association members.

Few men knew the serological status of their partner during last sexual intercourse (9% at last intercourse with a man and 2% with a woman). As respondent's HIV status, the partner's status as known by the respondent was not collected in the survey. Hence, it was not possible to connect condom use with the knowledge held by partners about their respective serological statuses. Neither can we determine possible partner selection strategies according to status. These strategies cannot be excluded however, since three anthropological interviews revealed a matrimonial strategy based on serological status [24].

A number of ideas can be drawn from our results for improving the existing prevention programs.

Firstly, it appears critical to insist upon the necessity of always carrying a condom. Not using a condom proves to be greater when sexual intercourse takes place outside or in a public place, where it is more difficult to get hold of a condom. However, while 60% of men surveyed declared that they always carried a condom, only 17% were able to actually show it to the investigator.

Bisexuality is extremely common among Senegalese MSM. Although informed individuals protect themselves more than others, unprotected sex during intercourse with a woman remains more common than during intercourse with a man. Also, condom use with a woman is no higher than within the rest of the Senegalese general population. But, female partners of MSM are more vulnerable due to high HIV prevalence among MSM. Therefore, programs targeting MSM must also take into consideration the heterosexual practices of this MSM population.

Lastly, while condom use is hardly appropriate with a wife when procreation is the reason for intercourse, screening of both partners should be highly recommended.

Whether intercourse takes place with a man or with a woman, the fact of having participated in a prevention program specifically targeting MSM constitutes a major prevention factor. This tends to show both the pertinence and effectiveness of these programs, and the necessity to pursue and to extend them.
